# Pharmacokinetics of Quinacrine Efflux from Mouse Brain via the P-glycoprotein Efflux Transporter

**DOI:** 10.1371/journal.pone.0039112

**Published:** 2012-07-02

**Authors:** Misol Ahn, Sina Ghaemmaghami, Yong Huang, Puay-Wah Phuan, Barnaby C. H. May, Kurt Giles, Stephen J. DeArmond, Stanley B. Prusiner

**Affiliations:** 1 Institute for Neurodegenerative Diseases, University of California San Francisco, San Francisco, California, United States of America; 2 Department of Biopharmaceutical Sciences, University of California San Francisco, San Francisco, California, United States of America; 3 Department of Pathology, University of California San Francisco, San Francisco, California, United States of America; 4 Department of Pharmaceutical Chemistry, University of California San Francisco, San Francisco, California, United States of America; 5 Department of Neurology, University of California San Francisco, San Francisco, California, United States of America; Biological Research Centre of the Hungarian Academy of Sciences, Hungary

## Abstract

The lipophilic cationic compound quinacrine has been used as an antimalarial drug for over 75 years but its pharmacokinetic profile is limited. Here, we report on the pharmacokinetic properties of quinacrine in mice. Following an oral dose of 40 mg/kg/day for 30 days, quinacrine concentration in the brain of wild-type mice was maintained at a concentration of ∼1 µM. As a substrate of the P-glycoprotein (P-gp) efflux transporter, quinacrine is actively exported from the brain, preventing its accumulation to levels that may show efficacy in some disease models. In the brains of P-gp–deficient *Mdr1* ^0/0^ mice, we found quinacrine reached concentrations of ∼80 µM without any signs of acute toxicity. Additionally, we examined the distribution and metabolism of quinacrine in the wild-type and *Mdr1* ^0/0^ brains. In wild-type mice, the co-administration of cyclosporin A, a known P-gp inhibitor, resulted in a 6-fold increase in the accumulation of quinacrine in the brain. Our findings argue that the inhibition of the P-gp efflux transporter should improve the poor pharmacokinetic properties of quinacrine in the CNS.

## Introduction

In the search for inhibitors of PrP^Sc^ formation, we and others found that phenothiazines and acridines like quinacrine lowered the levels of PrP^Sc^ in scrapie-infected neuroblastoma (ScN2a) cells [1], [2]. Those findings suggested the possibility that quinacrine might be an effective therapeutic for the uniformly fatal neurodegenerative disorder Creutzfeldt-Jakob disease (CJD) in humans. Disappointingly, quinacrine was ineffective in treating prion disease in humans and experimental animals [3], [4], [5]. The effective doses of quinacrine reducing PrP^Sc^ by 50% in cell cultures were reported to be ∼1 µM and ∼7 µM extracellularly and intracellularly, respectively [2], [6]. Puzzled by the lack of therapeutic efficacy, we examined the pharmacokinetic properties of quinacrine in mice.

Quinacrine is an acridine-based compound that was first synthesized in 1931 (Bayer, Germany) and initially used for the prophylaxis and treatment of malaria. Quinacrine has also been used to treat giardiasis, systemic lupus erythematosis and rheumatoid arthritis [7], [8]; it has also found utility as an anthelmintic [7] and female sterilization in a few countries [9]. Quinacrine also works against cutaneous leishmaniasis and Trypanosoma cruzi *in vitro* [10], [11]. As a nonselective inhibitor of phospholipase A2, quinacrine has thus been suggested as a possible therapeutic for neoplasms and heat-induced injury [12], [13]. Results from another *in-vitro* study suggested that quinacrine may be a potent compound for treating glioblastomas by inducing autophagic vacuoles leading to tumor cell death [14]. Quinacrine has been reported to stabilize p53, induce the upregulation of downstream pro-apoptotic molecules, and induce p53-dependent tumor cell death [15].

The delivery of quinacrine to the mouse brain is complicated by its rapid efflux via the P-glycoprotein (P-gp) transporter found in endothelial cells of the central nervous system (CNS) [16], [17], [18]. The P-gp transporter is well-studied in neoplastic cells and in the blood-brain barrier (BBB) [19], [20], [21], [22]. P-gp actively transports quinacrine across the BBB, inhibiting its accumulation and maintaining its concentration at ∼1 µM in the brain [23]. Therefore, the bioavailability and efficacy of quinacrine may be reduced when administered *in vivo* compared to effective concentrations determined *in vitro*. In this paper, we report on the pharmacokinetics of quinacrine in wild-type (wt) and *Mdr1* ^0/0^ mice in which the *mdr1a* and *mdr1b* genes encoding the P-gp transporters were ablated [24]. We established an oral administration protocol that maximized the accumulation of quinacrine, up to 200 µM, in the brains of *Mdr1* ^0/0^ mice without signs of toxicity. We also determined the half-life (t_1/2_) of quinacrine in the brains, spleens, kidneys, and livers of wt and *Mdr1* ^0/0^ mice. Using the intrinsic fluorescence of quinacrine, we were able to observe the anatomic distribution of quinacrine in the brains of quinacrine-treated mice. To increase the concentration of quinacrine in the brains of wt mice, we administered the P-gp inhibitor cyclosporine A (CyA), which produced a 6-fold increase in brain quinacrine levels.

## Materials and Methods

### Ethics Statement

All protocols involving animal studies were approved by the University of California San Francisco Animal Care and Use Committee (AN076161-02).

### Materials

Quinacrine dihydrochloride, acetonitrile, methanol, quinidine, CyA, verapamil, and disulfiram were purchased from Sigma-Aldrich (St. Louis, MO).

### Animals and Tissue Preparation

Approximately five-week-old male and female wt FVB and *Mdr1* ^0/0^ mice were purchased from Charles River (Wilmington, MA) and Taconic (Germantown, NY), respectively. For the orally treated group, mice were fed with 40 mg/kg/day of quinacrine in a chocolate-flavored liquid diet [25]. After treatment, mice (*n* = 3) were euthanized at various time points, and brain, spleen, liver, and kidney tissues were collected and flash frozen. Tissue homogenates (10% w/v) were prepared using a Precellys 24 homogenizer (Bertin Technologies, Montigny-le-Bretonneux, France) in pure distilled water (Invitrogen, San Diego, CA), aliquoted, and stored at −80°C.

### P-gp Inhibitor Study

P-gp inhibitor stock solutions of quinidine, CyA, verapamil, or disulfiram, were prepared in PBS or DMSO. The solutions were diluted to 100 mg/kg with PBS, based on 25 g of mouse body weight and the final injection volume of 200 µl per mouse. The P-gp inhibitor solution was administered to FVB mice (*n* = 4) by gavage. After 1 or 2 h of P-gp inhibitor administration, 10 mg/kg of quinacrine in PBS was given to FVB mice via gavage. As controls, *Mdr1* ^0/0^ mice were also treated with 10 mg/kg quinacrine in PBS via gavage. Mice were euthanized at 6 or 12 h post-quinacrine administration, and tissues were collected and flash frozen. Tissue homogenates (10% w/v) were prepared as described as above and 200-µl aliquots were stored at −80°C.

### Quinacrine Extraction

#### Preparation of standard solutions

Working solutions containing quinacrine, *O*-demethyl-quinacrine (M1), and mono-desethyl-quinacrine (M2) (10 µg/ml each) were prepared in 50% acetonitrile solution. Working solution was diluted with untreated, control 10% tissue homogenates to make 500 µl of 1 µg/ml of standard 1 solution for each tissue type. The standard 1 solution was then serially diluted two-fold. Nine standard solutions (200 µl each) including a blank solution (0 µg/ml) were frozen at −80°C. *Extraction.* A total of 400 µl of acetonitrile containing the internal standard (50 ng/ml of chlorophenamine) was added to 200 µl of 10% tissue homogenates or standard solutions. The samples were vortexed vigorously twice for 1 min and centrifuged at 16,000 *g* for 5 min.

### LC/MS/MS

Concentrations of quinacrine and metabolites M1 and M2 were measured by LC/MS/MS as described previously [18]. Briefly, the LC/MS/MS system consisted of Shimadzu LC-10 AD pumps, a Waters Intelligent Sample Processor 717 Plus autosampler, and a Micromass Quattro LC Ultima triple quadruple tandem mass spectrometer. The mass spectrometer was set to electrospray ionization in the positive-ion mode. Quinacrine, M1, and M2 were monitored by multiple reaction monitoring (MRM) at 400.5>142.2 *m/z* for quinacrine, 384.5>142.2 *m/z* for M1, 372.2>114.2 *m/z* for M2, and 277.2>142.2 *m/z* for internal standard (chlorphenamine). The column was a Betasil C18 column (50×4.6 mm) from Hypersil-Keystone and the mobile phase consisted of CH_3_OH/H_2_O/trifluoroacetic acid (45∶55∶0.05) with 1 mM ammonium formate. The flow rate was 0.8 ml/min.

**Table 1:** Quinacrine concentration (µM) in wild-type and *Mdr1* ^0/0^ mice.

Tissue	Wild-type	*Mdr1* ^0/0^	*P* value
Brain	1.6±0.3	84±2.4	0.0003
Kidney	55±17	138±33	0.01
Liver	207±25	223±50	0.7
Spleen	97±5.3	154±16	0.02

Wild-type and Mdr1^0/0^ mice (n = 3) were treated with 40 mg/kg/day of quinacrine in a chocolate-flavored liquid diet for 29 d. After treatment, the mice were euthanized and the quinacrine concentration in each tissue was measured by LC/MS/MS. Values are expressed as the mean ± standard deviation (SD).

**Figure 1:**
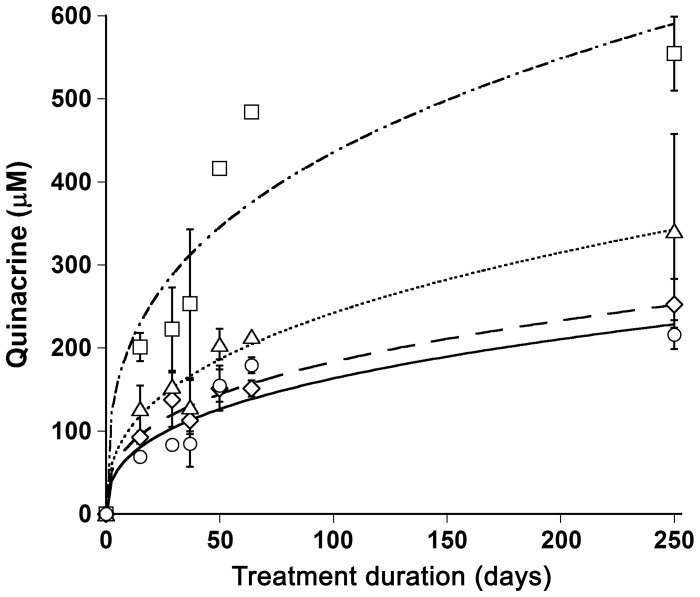
Quinacrine accumulation in brain (circles), spleen (triangles), liver (squares), and kidney (diamonds) tissues of *Mdr1* ^0/0^ mice. *Mdr1* ^0/0^ mice were treated with 40 mg/kg/day of quinacrine in a chocolate-flavored liquid diet continuously for up to 250 d. Mice were euthanized at the time-points indicated and the quinacrine concentration in each tissue was measured by LC/MS/MS. Data points are the mean concentrations (*n* = 3) and the error bars represent SD.

### Fluorescence Microscopy

Wild-type and *Mdr1* ^0/0^ mice were treated with 20 mg/kg of quinacrine via intravenous (IV) tail injection or 40 mg/kg/day of quinacrine in a chocolate-flavored liquid diet. At 1 and 24 h after quinacrine treatment, IV-injected mice were euthanized by CO_2_ asphyxiation and the cervical spine transected just below the skull. The brains were removed and snap frozen on powdered dry ice. Half of each brain was used for cryosectioning, and the other half for measurement of quinacrine concentrations. Frozen half-brains were mounted on a freezing stage using Cryo-gel (Instrumedics, St. Louis, MO) with the cerebellum facing out. Frozen coronal sections of 8 µm were cut at −22°C using a Microm HM 505E cryostat and mounted on Fisher Superfrost Plus slides. Serial sections were used for quinacrine and nuclear (4′,6-diamidino-2-phenylindole (DAPI), Vector Laboratories, Burlingame, CA) imaging. For quinacrine, frozen sections stored at −80°C were air-dried at room temperature (RT) for 10 min, mounted with 100 µl glycerin (Fisher, Hudson, NH), coverslipped, and sealed. When not on the microscope stage, slides were kept in a dark box on wet ice to slow disbursement of quinacrine in the tissue. All images were taken immediately after mounting using an inverted Leica DM IRB microscope through a FITC (fluorescein isothiocyanate) filter using a SpotFlex camera and Spot advanced software on the FITC setting. For DAPI, serial sections adjacent to those used for quinacrine imaging were air-dried at RT for 20 min and post-fixed in 4% formaldehyde (PolySciences Ultrapure EM grade, Warrinton, PA) in PBS for 15 min at RT, rinsed in PBS, mounted with Vectashield Mounting Media with DAPI, and imaged as described above, but using the DAPI filter and settings. Images were taken from the brainstem immediately adjacent to the most posterior portion of the cerebellum (Bregma −7.70 mm in the MBL c57Bl/6J Mouse Brain Atlas), at the interface of the granule cell layer and molecular cell layer in Crus II lobuli ansiformis and of the white matter track in the lobulus simplex midway through the cerebellum (Bregma −7.32 mm), of layer IV in the cortex immediately above the dentate gyrus, the dentate gyrus, and the thalamus immediately below the dentate gyrus (Bregma −2.212 mm). Image files generated on the Spot program were later processed in Adobe Photoshop for publication images.

**Figure 2:**
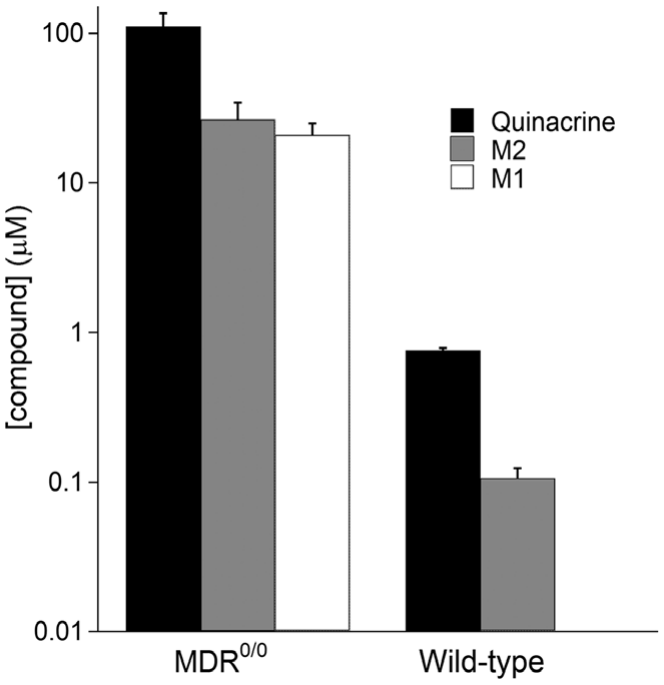
Steady-state concentrations of quinacrine and its metabolites in wt and *Mdr1* ^0/0^ mouse brains. Mice were treated with 40 mg/kg/day of quinacrine in a chocolate-flavored liquid diet for 31 d and then euthanized. The quinacrine concentration in the brain was measured by LC/MS/MS. Bars represent represent the mean concentration (*n* = 3); error bars represent SD. Y-axis shown in logarithmic scale. M1 metabolite levels for wild-type mice were under the detection limit.

**Figure 3:**
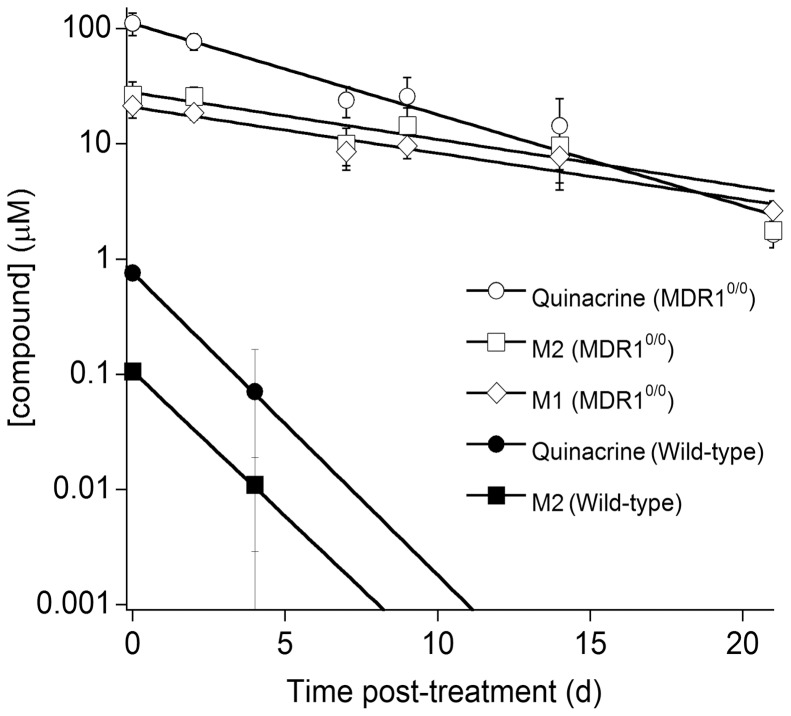
Clearance of quinacrine and its metabolites in wt (filled symbols) and *Mdr1* ^0/0^ (open symbols) mouse brains. Mice were treated with 40 mg/kg/day of quinacrine in a chocolate-flavored liquid diet for 31 d and then euthanized. The quinacrine concentration in the brain was measured by LC/MS/MS. Each data point represents the mean concentration (*n* = 3); error bars represent SD. Y-axis shown in logarithmic scale. Circles, quinacrine; diamonds, M1; squares, M2. Metabolite M1 levels for wt mice were under the detection limit.

## Results

### Accumulation of Quinacrine in the Brains of *Mdr1* ^0/0^ Mice

In earlier studies, we compared the levels of quinacrine in the brains of wt and *Mdr1* ^0/0^ mice after oral gavage or IV injections [18]. To study the therapeutic efficacy of quinacrine, we developed a chocolate-flavored liquid diet, in which quinacrine was dissolved [25]. In initial dosing experiments, we found that 40 mg/kg/day was the maximum tolerable and nontoxic dose. After feeding 40 mg/kg/day of quinacrine in a chocolate-flavored liquid diet for 29 days, quinacrine levels in brain, kidney, spleen, and liver tissues were measured by LC/MS/MS **(** **Table 1** **)**. In the brains of wt and *Mdr1* ^0/0^ mice, the quinacrine concentrations were 1.6 µM and 84 µM, respectively **(** **Table 1** **)**. The quinacrine concentration in the brains of wt mice was consistent with previously reported data [26]. *Mdr1* ^0/0^ mice showed higher quinacrine accumulation in the spleen and kidney than wt mice while the quinacrine concentration was similar in the liver for both wt and *Mdr1* ^0/0^ mice **(** **Table 1** **)**.

Next, we examined the quinacrine accumulation during long-term treatment. We treated *Mdr1* ^0/0^ mice with 40 mg/kg/day of quinacrine continuously for up to 250 d. Remarkably, quinacrine concentrations reached approximately 200, 250, 300, and 500 µM in the brain, kidney, spleen, and liver, respectively, with only minimal signs of toxicity **(** **Figure 1** **)**. In addition to discoloration of hair and/or skin, *Mdr1* ^0/0^ mice treated with quinacrine for 250 d showed some weight loss and reduced locomotor activity. Quinacrine concentrations in all four tissues rapidly increased after a few days of treatment and continued to increase gradually up to 250 d **(** **Figure 1** **)**. No quinacrine-induced neuropathology was observed in comparison to age-matched untreated brains. Mild focal necrosis and macrophage clusters were found in the livers of both treated and untreated mice (data not shown).

**Figure 4:**
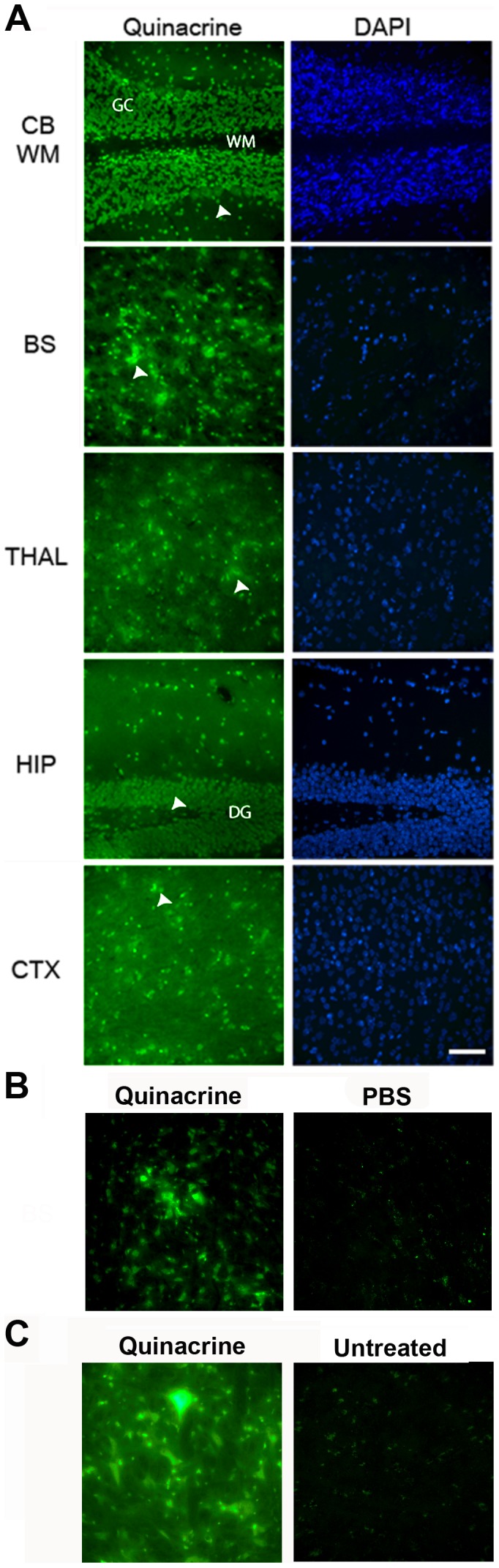
Distribution of quinacrine in the brains of *Mdr1* ^0/0^ mice by fluorescence microscopy. *Mdr1* ^0/0^ mice were treated with 20 mg/kg/day of quinacrine via IV tail-injection and euthanized after 1 h (**A**) and 3 h (**B**). In mice after 1 h, a high level of quinacrine fluorescence was seen in the nuclei of the cerebellum (CB) and hippocampus (HIP) as well as in the nerve cell bodies of the brainstem (BS), thalamus (THAL), and cortex (CTX). GC, granule cell layer; WM, white matter tract; DG, dentate gyrus; arrowheads, cell bodies of neurons or astrocytes. (**B**) In mice after 3 h, a high level of quinacrine fluorescence was observed only in the brainstem of quinacrine-treated mice (left) but not in the brainstem of PBS-treated mice (right). (**C**) Mice were treated with 40 mg/kg/day of quinacrine in a chocolate-flavored liquid diet for 25 days. A high level of quinacrine fluorescence was observed in the brainstem of quinacrine-treated mice (left), which was not observed in the brainstem of untreated mice (right). Scale bar in A represents 50 µm and applies to all images.

**Figure 5:**
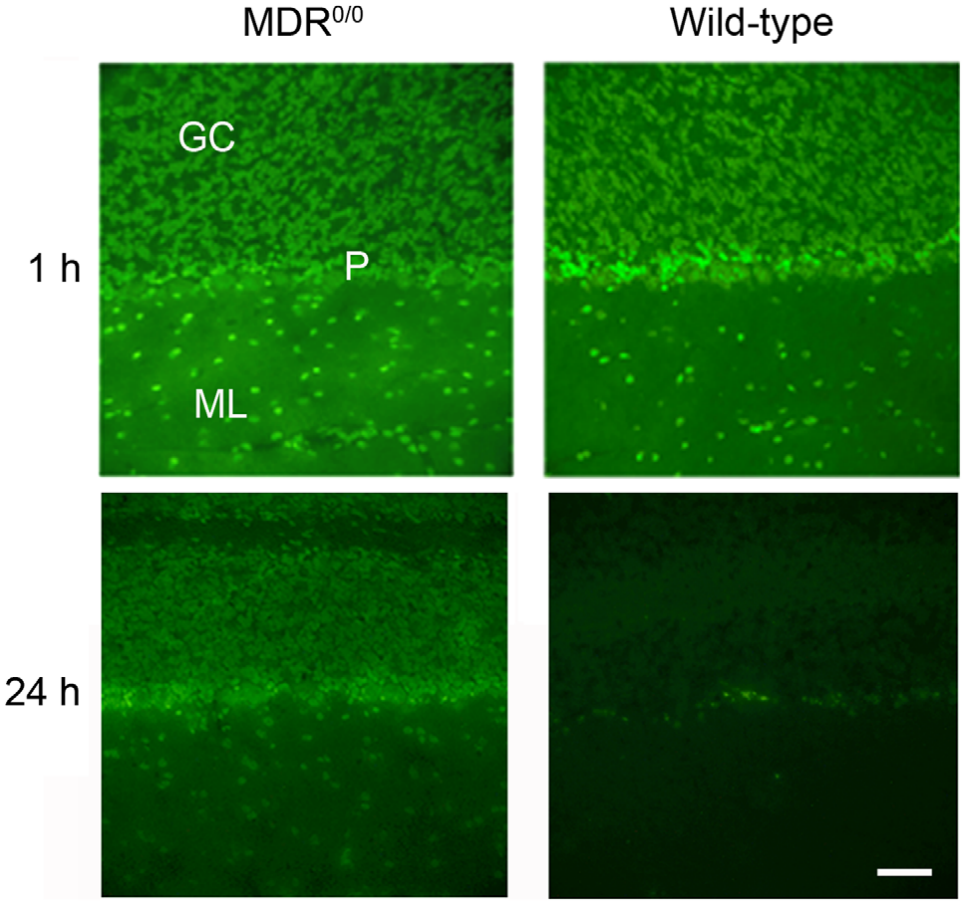
Distribution of quinacrine in the brains of *Mdr1* ^0/0^ and wt mice by fluorescence microscopy. Mice were treated with 20 mg/kg of quinacrine via IV tail-injection and euthanized after 1 h or 24 h. Brains with the highest signals for both wt and *Mdr1* ^0/0^ mice were chosen to get the best quality images, taken at 40×. At 1 h, quinacrine fluorescence was strong in both *Mdr1* ^0/0^ and wt mice. At 24 h, quinacrine fluorescence was observed only in *Mdr1* ^0/0^ mice; only lipofuscin autofluorescence in Purkinje cells was observed in wt mice. Scale bar represents 50 µm and applies to all panels. GC, granule cell layer; P, Purkinje cell layer; ML, molecular cell layer.

### Clearance of Quinacrine from the Brains of *Mdr1* ^0/0^ Mice

During the course of quinacrine exposure, the drug was metabolized by the processes of *O*-demethylation and *N*-desethylation to yield metabolites, *O*-demethyl-quinacrine (M1) and mono-desethyl-quinacrine (M2), respectively [18]. To examine the metabolism of quinacrine in the brain, we measured the concentrations of quinacrine and its metabolites in both wt and *Mdr1* ^0/0^ mice. Both wt and *Mdr1* ^0/0^ mice were treated with 40 mg/kg/day of quinacrine for 31 days. Tissues were collected at various time points after halting treatment and concentrations of quinacrine, M1, and M2 were measured by LC/MS/MS.

At the end of the treatment period, quinacrine and its metabolites (M1 and M2) achieved steady-state brain concentrations in both wt and *Mdr1* ^0/0^ mice. The concentration of M2, the major metabolite of quinacrine, was less than 25% of the quinacrine concentration in both wt and *Mdr1* ^0/0^ mouse brains **(** **Figure 2** **)**. We found a ∼100-fold difference in the brain quinacrine concentration between wt and *Mdr1* ^0/0^ mice **(** **Figure 2** **)**. In wt mice, quinacrine and its metabolites (M1 and M2) were rapidly cleared from brain by 7 d after the cessation of treatment; in contrast, quinacrine remained elevated in the brains of *Mdr1* ^0/0^ mice for up to 21 d after ceasing treatment **(** **Figure 3** **).** Notably, ∼1 µM brain quinacrine was found at the end of treatment phase in wt mice and 21 d after treatment ceased in *Mdr1* ^0/0^ mice **(** **Figure 3** **).** The concentrations of M1 for wt mouse brains were under the detection limit.

**Figure 6:**
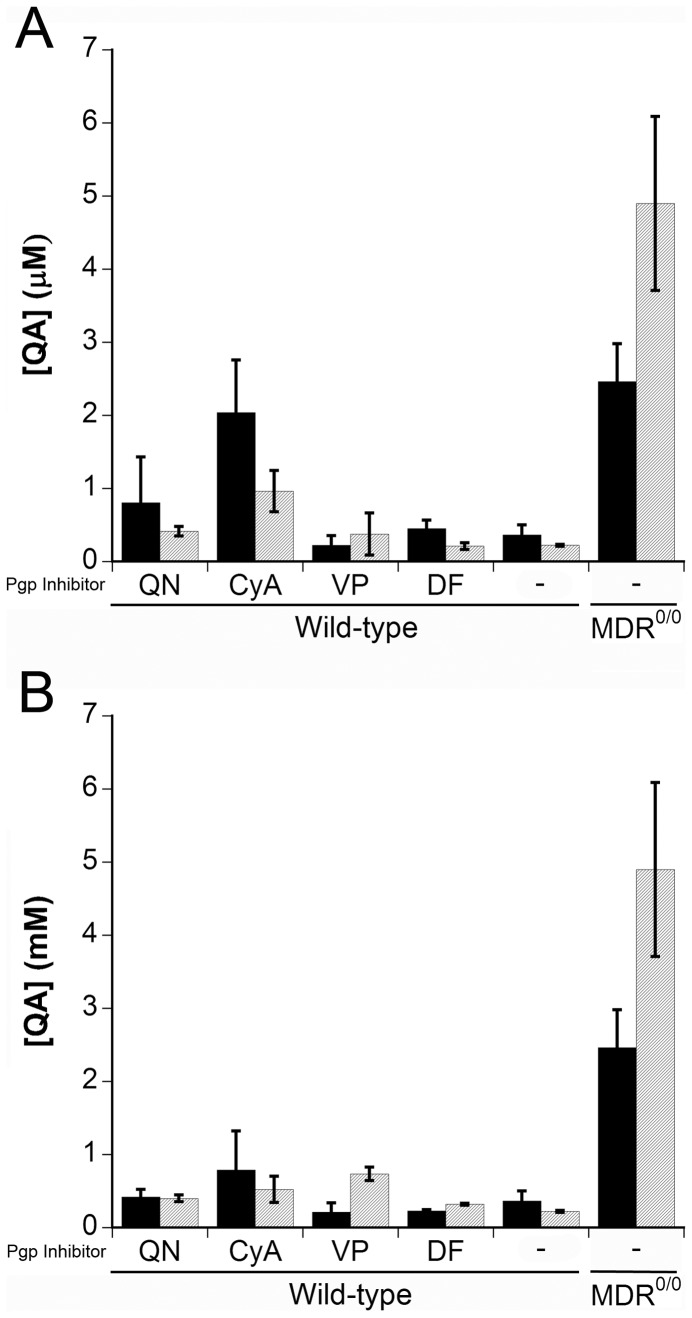
Quinacrine accumulation in the brain of the wt mice cotreated with different P-gp inhibitors and quinacrine. Wild-type mice (*n* = 4) were treated with 100 mg/kg of the indicated P-gp inhibitor by gavage; after 1 h (**A**) or 2 h (**B**), mice were treated with 10 mg/kg of quinacrine via gavage. Mice were then euthanized at 6 h (black bars) or 24 h (gray bars) after quinacrine treatment. As controls, wt and *Mdr1* ^0/0^ mice were treated only with quinacrine. The quinacrine concentrations were measured by LC/MS/MS. QA, quinacrine; QN, quinidine; CyA, cyclosporin A; VP, verapamil; DF, disulfiram; –, no P-gp inhibitor, QA only. Histograms are the mean concentrations (*n* = 4) and the error bars represent SD.

### Quinacrine Distribution in Mouse Brain

Having established that quinacrine accumulates and can persist for weeks in the CNS of *Mdr1* ^0/0^ mice, we examined its brain distribution using fluorescence microscopy. *Mdr1* ^0/0^ mice were treated either orally with 40 mg/kg/day of quinacrine for 25 d or by IV tail injection with 20 mg/kg of quinacrine for 1 h. The distribution of quinacrine in brain sections was visualized by imaging its intrinsic fluorescence. Quinacrine was clearly detectable in various regions of both orally treated and IV tail-injected *Mdr1* ^0/0^ mouse brains, but not in untreated and PBS-treated *Mdr1* ^0/0^ brains (**Figure 4B and 4C**). Although quinacrine was distributed throughout the brain, we observed differential distribution across brain regions. In the hippocampus, quinacrine fluorescence was intense in granule cell nuclei of the dentate gyrus **(** **Figure 4A** **)**. In the cerebellum, nuclear staining was observed in neurons and glia of the granule, Purkinje, and molecular cell layers, but little or no quinacrine fluorescence was seen in the white matter **(** **Figure 4A** **and** **5** **)**. The quinacrine signal was intense in nerve cell bodies of the cerebral cortex, thalamus, and brainstem **(** **Figure 4A** **)**.

In order to rule out the possibility of artificial localization of quinacrine due to the compositional changes in membranes resulting from the deletion of *mdr* genes, we also examined quinacrine distribution in the brains of wt mice. Although the quinacrine fluorescence signal was not detectable in the brains of wt mice treated orally with 40 mg/kg/day of quinacrine for 25 d, it was measurable when wt mice were injected with 20 mg/kg of quinacrine by IV tail-injection. At 1 h after IV tail injection, the quinacrine fluorescence signals in wt and *Mdr1* ^0/0^ were similar **(** **Figure 5** **)**. Notably, quinacrine signals were faint in the brains of wt mice collected at 3 h after IV injection (data not shown). By 24 h after IV injection, quinacrine signals were absent in the brains of wt mice but remained intense in *Mdr1* ^0/0^ mice **(** **Figure 5** **)**. These observations argue that wt and *Mdr1* ^0/0^ mice accumulate quinacrine in similar regions of the brain, but at different concentrations.

### P-gp Inhibitors Increased Quinacrine in Brains of Wild-type Mice

In order to be useful clinically, quinacrine levels in the brain must remain high, which can be achieved by co-administration of a P-gp inhibitor. We attempted to increase quinacrine levels in the brains of wt mice by utilizing four known P-gp inhibitors: quinidine, CyA, verapamil, and disulfiram. Groups of wt mice were treated with 100 mg/kg of the P-gp inhibitor for 1 h **(** **Figure 6A** **)** or 2 h **(** **Figure 6B** **)** prior to quinacrine dosing by oral gavage. Tissues were then collected 6 or 24 h after quinacrine administration.

Wild-type mice treated with 100 mg/kg of CyA prior to quinacrine showed a 6-fold increase in brain concentrations of quinacrine compared to controls **(** **Figure 6A** **)**, which was ∼70% of the concentration measured in the brains of P-gp–ablated *Mdr1* ^0/0^ mice after 6 h of quinacrine treatment **(** **Figure 6A** **)**. Quinacrine levels in CyA-treated mice only increased ∼3-fold when quinacrine was administered 2 h after CyA **(** **Figure 6B** **)** or when brain tissue was collected at 24 h post–quinacrine administration. Quinidine increased quinacrine in a similar pattern, but to a lesser extent **(** **Figure 6** **)**. In contrast, quinacrine brain concentrations were higher in the brains of wt mice receiving the P-gp inhibitor verapamil at 24 h compared to 6 h **(** **Figure 6** **)**. Also, lengthening the interval between verapamil and quinacrine administration to 2 h resulted in higher quinacrine concentrations in the brain **(** **Figure 6B** **)**. Administering a combination of different P-gp inhibitors may enable sustained increases in brain quinacrine levels. At 50 mg/kg doses, the P-gp inhibitors did not influence the quinacrine concentration in wt brain tissue (data not shown).

## Discussion

P-gp is a transmembrane glycoprotein that functions as an adenosine triphosphatase energy-dependent transporter. The BBB prevents many foreign substances, such as proteins, peptides, and most drugs, from gaining access to the CNS. P-gp is expressed in the endothelial cells of the BBB and functions as an active drug efflux pump. The P-gp pump keeps the concentrations of a wide variety of foreign substances low and thereby provides an important protective barrier against many potentially toxic molecules. P-gp also removes many drugs from the CNS [21], [24] and thus reduces their potential effectiveness [22]. This being the case, inhibition of P-gp may be required to increase the bioavailability of drugs targeting CNS diseases.

In a recent study, the utility of quinacrine in combination with a γ-secretase inhibitor cleared PrP^Sc^ from the brains of scrapie-infected mice [25]. Unfortunately, the survival of the scrapie-infected mice could not be prolonged due to toxicity of the γ-secretase inhibitor. Quinacrine also proved ineffective in mouse models of glioblastoma and heat-induced injury [12], [13].

In our study, P-gp inhibitors given prior to quinacrine administration increased the concentration of the drug up to 6-fold in wt mouse brain **(** **Figure 6** **)**. Although we have shown that inhibition of P-gp with CyA enhanced bioavailability of quinacrine in the brain, it is likely that the administration protocol is suboptimal to achieve maximal and sustained quinacrine concentrations in the brain for prolonged periods. Quinacrine was cleared from the CNS in several hours when it was administered with a P-gp inhibitor in wt mice. This observation suggests that higher or frequent doses of quinacrine with a P-gp inhibitor may need to be given. In our study, CyA seemed to be a fast-acting inhibitor while verapamil was a slow-acting inhibitor: quinacrine concentrations were highest at 6 h with CyA and at 24 h with verapamil **(** **Figure 6** **)**. In future studies, it might be interesting to examine combinations of P-gp inhibitors.

Since its synthesis in 1931, quinacrine has been widely used for treatment and prevention of malaria and chronic inflammatory conditions [8]. The mechanisms of action of quinacrine remain unclear but many recent studies have suggested that it might be useful in treating cancer and neurodegenerative diseases. In addition to its ability to intercalate into DNA, quinacrine has been reported to regulate transcription factors, including NF-κB, hsp70 and p53 [12], [27], [28], which control immune responses, proliferation, and tumor genesis. Another study suggested that quinacrine protects heat-induced neuronal injury via inhibition of cPLA2 [13]. Interestingly, a multimeric quinacrine conjugate has been reported to inhibit Aβ-amyloid fibril formation [29].
